# “Pick up anything that moves”: a qualitative analysis of a police crackdown against people who use drugs in Tijuana, Mexico

**DOI:** 10.1186/s40352-020-00111-9

**Published:** 2020-04-29

**Authors:** Mario Morales, Claudia Rafful, Pieter Baker, Jaime Arredondo, Sunyou Kang, Maria L. Mittal, Teresita Rocha-Jiménez, Steffanie A. Strathdee, Leo Beletsky

**Affiliations:** 1 School of Government and Public Policy, University of Arizona, Tuscon, USA; 2 Faculty of Psychology, Universidad Nacional Autónoma de Mexico, Mexico City, Mexico; 3 Center for Global Mental Health Research, National Institute of Psychiatry, Mexico City, Mexico; 4 Centre for Urban Health Solutions, St. Michael’s Hospital, Toronto, Canada; 5 Division of Infectious Diseases and Global Public Health, School of Medicine, University of California, San Diego, 9500 Gilman Drive, La Jolla, CA 92093-0507 USA; 6 School of Public Health, San Diego State University, San Diego, USA; 7 School of Law & Bouvé College of Health Sciences, Northeastern University, Boston, USA; 8 School of Medicine, Universidad Xochicalco, Tijuana, Mexico; 9 Society and Health Research Center, Facultad de Humanidades, Universidad Mayor, Santiago, Chile

**Keywords:** Mexico, People who use drugs, Police officers, Drug law enforcement, Crackdown, Involuntary drug treatment referral

## Abstract

**Background:**

Homeless people who use drugs (PWUD) are often displaced, detained, and/or forced into drug treatment during police crackdowns. Such operations follow a zero-tolerance approach to law enforcement and have a deleterious impact on the health of PWUD. In Mexico, municipal police officers (MPOs) conducted the largest crackdown documented at the Tijuana River Canal (*Tijuana Mejora*) to dismantle an open drug market. We analyzed active-duty MPOs’ attitudes on the rationale, implementation, and outcomes of the crackdown. We also included the involvement of non-governmental allies in the disguised imprisonment as drug treatment referral and potential legal consequences of having illegally detained PWUD.

**Methods:**

Between February–June 2016, 20 semi-structured interviews were conducted with MPOs in Tijuana. Interviews were transcribed, translated and coded using a consensus-based approach. Emergent themes, trends and frameworks were analyzed through a hermeneutic grounded theory protocol.

**Results:**

Participants recognized the limitations of *Tijuana Mejora* in effectively controlling crime and addressing drug treatment solutions. MPOs perceived that the intent of the operation was to displace and detain homeless PWUD, not to assist or rehabilitate them. The police operation was largely justified as a public safety measure to reduce the risk of injury due to flooding, decrease drug consumption among PWUD and protect local tourism from PWUD. Some participants perceived the crackdown as a successful public health and safety measure while others highlighted occupational risks to MPOs and potential human rights violations of PWUD.

**Conclusions:**

*Tijuana Mejora* illustrated why public and private actors align in enforcing zero-tolerance drug policy. Perceptions of care are often based on captivity of the diseased, not in health and well-being of PWUD. Officer perceptions shed light on the many limitations of this punitive policing tool in this context. A shift towards evidence-based municipal strategies to address drug use, wherein police are perceived as partners in harm reduction rather than antagonists, is warranted.

## Background

Between December 2014 and March 2015, law enforcement in Tijuana, Mexico, banished approximately 1000 individuals living along the Tijuana River Channel (*El Canal*). The *Tijuana Mejora* crackdown to dismantle an open drug market in the US-Mexico border was a concerted police operation that included all levels of government: municipal, state and federal. It managed to lock up most of the evicted individuals into local drug centers^1^ for several months. (Rafful et al., 2019). Given prior failed attempts to “clean” *El Canal (*Albicker & Velasco, 2016*)*, we aimed to understand unique perspectives of this police operation. Specifically, we were interested to learn from the municipal police officers (MPOs) about their perceived attitudes and perspectives on the rationale, implementation, and outcomes of *Tijuana Mejora*. Likewise, we wanted to explore MPO reactions of and interactions with homeless people who use drugs’ (PWUD) related to the crackdown. These perspectives are important for understanding police crackdowns and zero-tolerance policing strategies against PWUD as they cause a variety of deleterious effects among this vulnerable population.

### Zero-tolerance and police crackdowns

A zero-tolerance policing strategy includes the intense law enforcement of petty crime to prevent real crime. It involves taking informal-extralegal steps to redefine public order (Kelling & Wilson, 1989). The rationale is that “if a window in a building is broken and is left unrepaired, all the rest of the windows will soon be broken” (Kelling & Wilson, 1989). The term “zero-tolerance policing” is associated with a specific set of policing strategies adopted in New York during the 1990s (Bowling, 1999; Greene, 1999), however, it has a lengthy history (Newburn & Jones, 2007). In the XVII century, France created a General Hospital to incarcerate poor people and get rid of societal problems such as begging and crime (Chamayou, 2012). In the XXI century, zero-tolerance policies endured in response to the rise of social, economic, and political insecurities created by economic deregulation and social-welfare retrenchment (Stuart, 2015; Wacquant, 2001).

Crackdowns are one specific strategy of zero-tolerance policing (Coomber, Moyle, & Mahoney, 2017). They involve the sudden increase in police threats, sanctions, and arrests for general or specific offences in particular places, for example, drug consumption in open drug markets (Ediomo-Ubong, 2018; Mazerolle, Soole, & Rombouts, 2007; Polomarkakis, 2017). Anti-narcotic crackdowns are supported by the UN Conventions of 1961, 1971, and 1988, which sanction the prohibition and criminalization of drug possession, use, and manufacturing (Hughes & Stevens, 2010). They aim to reduce and disrupt both drug supply and demand by increasing the risk of arrest and incarceration for sellers and buyers (May & Hough, 2001; Shepard & Blackley, 2005). They also prioritize punishment over rehabilitation under the assumption that PWUD respond to the criminal justice system’s deterring incentives (Polomarkakis, 2017; Stuart, 2014).

Several systematic evaluations have found negative outcomes of such strict drug law enforcement (Mazerolle et al., 2007; Sherman, 1990), including the appearance of new players in the drug market (Polomarkakis, 2017) and the marginal effect of seizures on drug price, purity, and availability (Caulkins, 2002; Wood et al., 2004). Additionally, there is consistent evidence on the positive association between crackdowns and geographical displacement of crime and drugs. This significant association harms the quality-of-life and public health in surrounding areas by increasing the risk of infectious disease transmission, drug overdose, and other drug-related harms (Kerr, Small, & Wood, 2005; Small, Kerr, Charette, Schechter, & Spittal, 2006). Moreover, authorities have attempted to justify crackdowns as a harm reduction strategy wherein drug treatment may be coerced. As such, police operations often force PWUD into drug treatment in a way that is often involuntary and in violation of human rights principles (Dixon & Maher, 2005).

A renewed consensus has emerged to promote a balanced approach to drug policy based on both public security and public health strategies (Caulkins, 2002; Goetz & Mitchell, 2006). This approach combines harsh punishment of drug traffickers and dealers along with the promotion of drug treatment and harm reduction strategies among PWUD (Cohen & Csete, 2006). In this context, law enforcers are encouraged to stop being “rabble managers” and become “recovery managers” (Stuart, 2014). The aim is to promote police officers’ direct role in rescue operations (e.g., using naloxone, which reverses opioid overdoses) (Beletsky, Rich, & Walley, 2012), referral to evidence-based harm reduction services (e.g., syringe exchange programs, methadone maintenance therapy, and safe injection facilities) and drug treatment centers (Beletsky, Macalino, & Burris, 2005; Hunter, McSweeney, & Turnbull, 2005; Watson et al., 2012), the employment of Good Samaritan practices (i.e., amnesty for witnesses who call for help in case of an overdose) (Banta-Green, Beletsky, Schoeppe, Coffin, & Kuszler, 2013), and the dissemination of information to reduce drug-related problems (Beletsky, 2016). This approach depends heavily upon the availability of such public health and harm reduction resources, as well as the political will to de-escalate from a zero-tolerance strategy. While this more balanced approach has become favored in certain settings of developed cities, PWUD in Tijuana continue to experience harms associated with zero-tolerance policing. Tijuana is considered an international benchmark to assess the implications of drug law enforcement in the context of the war on drugs (Brown, 2018; Osorio, 2015).

### Drug law enforcement in Tijuana, Baja California, Mexico

Tijuana, located along the US-Mexico northwest border, was barely recognized as a municipality in 1954 (Piñera & Rivera, 2013) but is now considered the busiest land border crossing in the world (Brouwer et al., 2011). Today, crime prevention is a primary concern as violence has increased over the past few decades due to inequality, economic challenges, organized crime competition, the war on drugs, and the enforcement of migration and drug policies in the U. S (Shirk, 2014). Enhanced law enforcement operations have been implemented over the years in response to the increase in crime, but they have had limited positive effects. In January, 2007, the federal government began a joint military-police operation to confront organized crime at the state and local levels (Tijuana Operation) (Ortega-Granados, 2017). About 3500 law enforcers participated in the intervention as a military chief became in charge of reorganizing the Tijuana Police Department and military units were incorporated in the police force (Contreras-Velasco, 2017; Shirk, 2014). However, Baja California’s homicide rate per 100,000 inhabitants increased from 12 in 2007 to 32 in 2016 and Tijuana constituted around 70% of the homicides in the state (INEGI 07/26/, 2017). Additional problems arose when the Lieutenant-Colonel responsible for reorganizing the Tijuana Police Department was suspended for 8 years after 25 municipal police officers (MPOs) accused him of torture, imprisonment, and ties to organized crime (Contreras-Velasco, 2017).

In 2009, Mexico passed national *Narcomenudeo* drug law reforms to integrate a health-based approach on drugs (DOF, 2014). However, they have not been effectively implemented in the city because the municipal police had prioritized the enforcement of local quality-of-life ordinances, which criminalize behaviors associated with homeless PWUD, such as loitering (Morales et al., 2020). This misalignment of policing practices and public health priorities has had deleterious consequences for PWUD in Tijuana. In 2013, it was reported that arrests leading to criminal charges among PWUD increased from 42.4% in 2011 to 89.6% in 2013 (Gaines et al., 2017). PWUD have also reported that drug law enforcement increased their risk behavior for blood-borne infection transmissions by reducing their willingness to carry sterile syringes and personal injecting equipment, and increasing receptive needle sharing, rushed injection, and shooting galleries attendance (Beletsky et al., 2013; Volkmann et al., 2011). Places where PWUD (including those with HIV) used to live were relatively static until July 2008, when there was a dispersion to southeast Tijuana. This potentiated the dispersal of the drug market and the transmission of blood-borne infections (Brouwer et al., 2012). PWUD have also reported increasing human rights violations as a result of police harassment (e.g., verbal and physical abuse, and payoffs) and involuntary police referrals to drug centers, which have accompanied crackdowns in August 2013 and March 2015 (Contreras-Velasco, 2016; Rafful et al., 2019).

Police crackdown operations continue in Tijuana, despite ample evidence that zero-tolerance policing has done nothing to curb violent crime and has resulted in elevated harm to PWUD and other vulnerable groups (Velasco & Albicker, 2013). Previous research has focused on the pervasive police violence against PWUD in Tijuana from the perspective of the victims (Albicker, 2014; Pinedo et al., 2015; Wood et al., 2017). However, this study is innovative in its focus on drug law enforcement from the perspective of MPOs, contributing to the emerging literature about officers’ attitudes and behaviors on drug law enforcement (Contreras-Velasco, 2017). Moreover, this study describes how governmental and non-governmental agents cooperate to involuntarily refer PWUD to drug centers. For the purpose of informing drug policy reform efforts, public health interventions, and law enforcement practices in Mexico and abroad, this qualitative study examined the design, implementation, and MPOs’ self-evaluation of a crackdown at El Canal in Tijuana from December 2014 to March 2015.

## Methods

This study was conducted as part of the SHIELD project, a police education program intended to assess drug law enforcement in the context of blood-borne infection prevention (e.g., HIV, HBV, and HCV) (Strathdee et al., 2015). Participants were recruited from the Tijuana Police Academy, out of which approximately 770 MPOs consented to complete pre-, post-training and follow up surveys (Strathdee et al., 2015). All of them provided written consent to be contacted for the qualitative sub-study. As part of the inclusion criteria for the qualitative arm, all respondents were active-duty, completed SHIELD training and baseline survey before the interview, and reported ever experiencing a needle stick injury on-duty (one respondent expressly requested to participate without meeting the last two criteria).

Interviews took place across Tijuana from February to June 2016 at the participants’ convenience. The main themes covered in the interviews were MPO’s occupational safety; MPO’s decision-making process to detain illicit drug carrier suspects; the presentation of PWUD before judges at the municipal, state, and federal levels; and referral of PWUD to treatment services. Participants were encouraged to elaborate on their experience of *Tijuana Mejora*. Once we reached 20 interviews, the narratives reached saturation and became repetitive and we stopped conducting more interviews (i.e., saturation of main topics). The interviews lasted from one to two hours and were all conducted by the first author. They followed a face-to-face, in-depth, and semi-structured format, in which the first author followed an interview guide, took detailed notes during the interview, and wrote personal impressions of the interview in a fieldwork diary. Respondents were guaranteed confidentiality and anonymity and were provided $20 incentive in the form of a movie theater gift card as compensation.

Interviews were audio-taped, transcribed verbatim and translated from Spanish to English. Transcriptions were verified against the audio records and translations were double-checked for quality by trained bilingual personnel. A hermeneutic grounded theory approach was used to analyze the data and to explore the individual production of meanings and concepts in real settings (Charmaz, 2000; Glaser, 2004; Strauss & Corbin, 1994; Suddaby, 2006). Atlas.ti software was used to create hermeneutic units: working documents (i.e., transcripts), quotes (i.e., segments of transcripts), codes (i.e., basic grouping units of analysis), annotations (i.e., theoretical-methodological reflections) and code families (i.e., group of codes) (Avalos & Utley, 2014). Following an inductive and iterative approach, we read each transcription to identify the most significant sentences for the subject of study (i.e., exploration). Then, we tagged keywords and defined concepts to encompass the main elements of each significant statement (i.e., codification). We also adjusted the three categories of codes (i.e., theoretical, practical and lingual) to facilitate subsequent formation of analytical categories (i.e., families). During the process, we discussed emerging keywords, concepts and families, and their inter-relationships until discrepancies were resolved. This study had binational ethical approval from the Human Research Protections Program of the University of California, San Diego in the United States and the Institutional Review Board at Universidad Xochicalco in Tijuana, Mexico.

## Results

Out of the 20 participants, most (*n* = 15) were men and officers (as opposed to being district chiefs, deputies, or supervisors). They were aged between 22 and 63 years old (median age 38). Participants were aware that *Tijuana Mejora* was a controversial action and 65% argued that they did not participate in it. However, all were active MPOs at the time of the crackdown and expressed personal percpetions and attitudes regarding the police operation. Four main findings emerged from the analysis. First, participants justified the crackdown as prevention of social damages associated with floods (i.e., accidents and deaths) and as promotion of drug treatment, public security, and tourism. Second, MPOs characterized the crackdown as a large, coordinated, scary (for both MPOs and PWUD), and rushed operation that was preceded by efforts to displace people from El Canal such as free transportation to their places of origin, intensification of drug law enforcement, and referral to shelters or drug centers. Third, MPOs perceived the outcomes of the crackdown as both prevention of crime and traffic accidents (because PWUD were regularly hit by cars at major highways) as well as a means to lower homeless visibility. Fourth, MPOs shared attitudes on how PWUD are systematically imprisoned in the municipal jail for quality-of-life offences. Many questioned the voluntary nature of the drug centers’ referral that followed the crackdown and expressed concern about being accused of kidnapping PWUD.

### Perceived rationale for the crackdown in El Canal

Participants stated that El Canal banishment was promoted at all levels of the government with the primary rationale of avoiding deaths during the coming raining season and to refer people to drug centers.

Because the Tijuana River Canal is federal property, the federal government asked us [MPOs] for support in convincing the people to get out of El Canal prior the heavy rains … They say: let’s get all the people out of El Canal so they can stay out of their vicious cycle. El Canal would eat them up and all they would end up in drugs (Man, 28).

They explained it to us like that. You know what? We need to convince the largest amount of people possible that live at El Canal to leave. The rains are coming, um … we need to get them out of there. Why? Because it’s federal property, they said, the federal government is asking us for support in getting those people out of there, to convince them, um, and to send those who want to go to rehabilitation. We must convince the most people possible, so they go to a rehab center and get rehabilitated. And that was pretty much how they told us and … we just obey orders here […] I think treatment was going to cost 5000 pesos per person [approximately 250 USD], and between the local and federal governments, I don’t know how much each, were going to pay the rehabilitation center. The rehabilitation center then gave people free treatment (Man, 28)*.*

Respondents also mentioned that it was necessary to clean the area to improve public security and aesthetics for the sake of local commercial interests and tourism.

Businesses started to see more people from El Canal in the areas where there is more tourism and that created a bad image to the city … I consider it was really necessary to clean up the area. El Canal was not built so people could live there, especially with these types of problems. Any heavy rain and they were under big risk of maybe even dying … What they [businesspeople] asked for was more security and vigilance in those areas (Man, 38).

MPOs pointed out the need to move the greatest number of PWUD from areas with high crime rates to spaces of protection and rehabilitation. However, at no time did they provide evidence to support that shelters and drug centers are safe spaces, which is typically not the case in Mexico and Central America. Several studies have criticized these centers by the lack of state regulation, non-evidence based approaches to rehabilitation, a religious for-profit business model and forced captivity (O'Neill, 2019; Rafful et al., 2019). Consequently, for some MPOs, it was hard to believe that the main aim of the crackdown was to safeguard people living in El Canal.

### Officer experiences and perspectives on police tactics to banish people from El Canal before and during the crackdown

### Population displacement

At the onset of the crackdown, social services authorities placed tents in El Canal and invited the people living there to go back to their places of origin, to get into shelters, or to admit themselves at drug centers:

At the beginning the city council set up many tents in El Canal. First, they offered support for those [who wanted] to go back to their cities of origin or [to go to] rehabilitation centers. [Then, when] the government considered that the time was up and it started kicking out all those people [from El Canal] (Man, 33)*.*

Municipal judges and drug center staff also invited PWUD to enter drug centers while they were in the municipal jail due to quality-of-life ordinances (e.g., drinking alcohol, using drugs or sleeping/urinating in public places). However, according to most MPOs, not all judges wanted to refer people to drug centers and most PWUD did not want to be referred. Some MPOs also noted the discretionary selection of the drug centers that were partnered with the government to receive PWUD from the crackdown.

“The judge would ask them if they were addicts and if they wanted rehabilitation. Those who say yes signed a paper and went to rehabilitation centers voluntarily. We transferred them to the rehabilitation centers and they walked in on their own … There were a lot of drug centers: CIRAD, CRREAD, *Mesón*. A lot of them were affiliated so they could get local government support” (Man, 33)*.*

“A group of people from the drug centers used to go to [the municipal] jail, and before putting [PWUD] in the cells or before handing them to the guarding officers from jail … they told them that those who wanted to recover … were going to be admitted [in the drug center] … They were going to have food, clothes, heat, all the support. Some did accept, like four or five among 30 to 50” (Man, 46)*.*

Several people who lived in El Canal accepted the offer of going back to their places of origin. However, it is likely that not all had the opportunity to do so. One participant criticized the local government for not fully paying the travel tickets and for not providing enough buses to transport the people living in El Canal to their places of origin.

“There were some that wanted to go [back to their places of origin] and only got half of the bus fare, they didn’t have a way to pay for the other half, even if it wasn’t a lot of money … There was something else: they sent very few buses … I’m not so sure, but I think it was like two hundred people … It was about six or eight buses that left from Padre Chava’s [Salesian Refectory] to different places” (Woman, 37).

MPOs recognized that both governmental and non-governmental actors provided attention to PWUD prior the crackdown. It is evident that there are different techniques to track and capture PWUD. Consequently, the following questions arise: is there a formal collaboration between municipal authorities and drug centers to conduct referrals? In what terms is the agreement defined? What is the process to allocate government resources into drug centers? If it is true that only half the cost of travel tickets was paid, why and who decided to give this type of support to PWUD?

#### Intensification of drug law enforcement

The municipal government abruptly escalated drug law enforcement activities along El Canal to increase the perceived and actual threat of apprehension for quality-of-life ordinances. This was used to motivate people to displace themselves.

“The first week [of *Tijuana Mejora*] many of them [PWUD] didn’t choose to go to rehabilitation centers, so we sent them to the municipal jail. The second week, they saw that they wouldn’t be able to handle us [because] we don’t stop until we accomplish our objective [Canal banishment]. I said: you’re going to go to jail every day, so it’s better if you go to a rehabilitation center. We also got some drug distributors and all the addicts there were left without someone to provide them with their drugs” (Man, 28).

This demonstrates that MPOs perceived a specific goal, to remove all people from El Canal. Likewise, referral to drug centers were not optional for PWUD as it became mandatory. Compared to the local jail, captivity in drug centers was more appealing because more PWUD could be stored there.

#### Crackdown

Respondents agreed that in March 1, 2015 about “1,000 people were detained and 500 to 700 went to drug centers” (Men, 28 and 41). One participant quantified that “there were 20 to 30 officers from each police district” (Woman, 34) or, approximately 275 officers from 11 districts*.* Another respondent indicated that “about 400 law enforcers and 130 police units participated in all the operation” (Man, ~ 40).

The crackdown had three main features. First, a coordinated and quick accesses blockade strategy was implemented to avoid people getting out of El Canal*.* A human barrier was created to close all the access to El Canal and to sweep the area from south to north to congregate all the people along the US-Mexico border line.

“We [MPOs] arrived at three in the morning when they [PWUD] were sleeping … We blocked all the accesses to El Canal [with patrol units] and the commercial police officers stood at the top [of El Canal]. Even the Border Patrol blocked off the part of the river that was on their side [US] … Some colleagues in plainclothes were up on the bridge and discreetly gave the order: “go in!” Traffic [at the highways] had been stopped already by transit officers and we all ran in [to El Canal]. It began in *Zona Río* and it finished up to the border [~ 4 km], that’s where everyone came together” (Man, ~ 40).

Secondly, during the crackdown, both PWUD and MPOs were concerned for their personal safety*.* PWUD did not know what was going on and MPOs did not know what they would find in El Canal (especially upon entering El Canal’s storm drain tunnels). Moreover, MPOs did not follow occupational safety protocols while they were frisking PWUD, which increased their exposure to blood-borne infections.

“We flashed our light at people so they started waking up. We also got them out of the manholes and we took manholes down. We lined them up and we frisked them against the patrol units … Some were scared or upset, and others knew what was going on. Only the ones who got into the tunnels could escape because none of us went into the tunnels … I wasn’t going to pressure myself into getting him out of such a horrible place, right? Most of us did not use protection [gloves] because it wasn’t appropriate for what we were doing … [but] we did find *globos* [meth doses] and needles” (Woman, 34).

Lastly, the municipal judges and drug centers’ staff streamlined the referral process*.* Reportedly, municipal judges and drug centers’ staff were also present in El Canal to speed up the referral process to shelters, drug centers, or the police stations. Some MPOs elaborated on the due process, or lack thereof, emphasizing that PWUD had to be voluntarily admitted into the centers.

“Even municipal judges were taken out of their police stations and went to El Canal. Of those who ended their shifts in the morning, some were told to stay [for another shift]. The municipal jail also was prepared because it must be empty so that all those people fit there, that day it was at maximum capacity … There were also different rehabilitation centers’ staff in El Canal and it was voluntarily [referral process]. Remember they [PWUD] can’t go against their will [to drug centers] … Those who didn’t [accepted], well, it was the same recycling system: they were detained, they were thrown into the county jail for a few hours and they went out” (Man, ~ 40).

*Tijuana Mejora* required the participation of all local law enforcers and the support of state and federal police agencies. It also demanded the special collaboration of municipal judges and drug center staff in the field. The compulsory nature of the operation implied the prevalence of MPO’s occupational safety risks and PWUD’s human rights violations. Likewise, judges conducted deficient decision-making processes due to the high demand of work in a short period of time.

#### Perceived consequences of the crackdown in El Canal

Participants reported perceived reductions in crime, car accidents in the two main highways in the area, and the MPOs’ ability to locate potential criminals out of El Canal.

“Now, it is a little bit less problematic around El Canal. There were a lot of house theft, shoplifting and accidents on the highway, and they decreased” (Man, 33).

“It is a little bit more crowded up here [Downtown]. Those who slept there [El Canal] are sleeping over here now … I think it’s easier for us than before because they used to steal, run to El Canal and get lost inside the sewers, right? And now you can locate them here, you locate them more easily” (Man, 33).

However, respondents were suspicious about the effectiveness of the crackdown, particularly in terms of public image, one of the aims of the crackdown. MPOs talked about the negative “cockroach effect”, where people living in El Canal moved to other places to continue committing quality-of-life ordinances.

“The changes were merely visual. Now you don’t see them when you’re driving on the highways [*La Vía Rápida Poniente* and *La Vía Rápida Oriente*]. They were looked like ants before, now they are spread apart. It’s like, you took a problem and moved it from here to other place” (Woman, 34).

“People need a place to stay, to sleep, to eat, to buy drugs, so they went towards the surroundings and the crime rate increased in those places … Now small-traders have people sleeping outside their business and messing with the trash containers. They became a nuisance and now we must carry out police operations to take them away from those places … We have to arrest them and bring them into the municipal jail” (Man, 30).

Because of the crackdown in El Canal, MPOs reported a decrease in crime perception in the area, but an increase of it in the perimeter. A participant mentioned that now PWUD are more visible in the Downtown area and it is easier for MPOs to deal with them individually, rather than as a mass of individuals living in community. Nonetheless, other respondents acknowledged that Tijuana is far from solving the problem of homelessness and drugs because now MPOs must conduct more police operations to dissuade PWUD from being in the Downtown area.

#### Attitudes toward involuntary drug treatment referral after the crackdown in El Canal

MPOs discussed their attitudes on incarcerating PWUD in a daily basis to enforce quality-of-life ordinances.

There are areas where I can search 10 people, but there are other areas where I can search about 30 people [in a 12 h-shift]. Here in Downtown … mostly recyclable people, that’s how we call them. People who are in and out. We bring them in [the police station] for drug consumption, we put them in front of a judge, they go to the public jail, they get out and we get them again in the same area … We coexist with them and we get to know them. For example, I say: ‘Ey, what are you doing here?’ And they will tell me: ‘Ok boss, I’m leaving.’ And it’s like: ‘Go on, get out of here, get out of my work area.’ If they don’t listen: ‘sorry but you have to come with me to jail’. (Man, 39).

Additionally, involuntary drug treatment referral has emerged as a new mechanism to lock up PWUD. However, all but one participant perceived that voluntary drug treatment was better than involuntary drug treatment for substance use disorders.

*“‘A la fuerza ni los zapatos entran’* [nothing can be successfully accomplished by force]... You have to talk to them [PWUD] and try to convince them to get psychological and drug treatment” (Man, 41).

One participant, who reported not having participated in the crackdown, mentioned that he would prefer to be arrested for breach of duty than to be forcing people into drug centers. He also mentioned a family member of his had been in drug centers and he knew that they operate in poor conditions. For him it was not only about breaking the law, but about having empathy with the people living in El Canal.

“I think they [authorities] made a mistake. Instead of kicking them out [of El Canal], why didn’t they help them? There should be an area for them, so they can start rehabilitation. They have nowhere to go … I didn’t participate in the operation and, if I had had to, I would have refused to be honest. I prefer to be arrested rather than to be doing something that’s not right” (Man, 26).

In contrast, one MPO demanded citizens’ unconditional support to refer PWUD to drug centers, no matter human right violations.

“It should be mandatory to be referred to drug centers if you are brought in for drug use … Sorry, but you’re already a problem to society. It’s not a matter of whether you want to or not, you should be checked into a center” (Man, 33).

Considering most participants expressed antipathy to forced treatment referral and fear of being accused of kidnapping PWUD, we should focus on those who are planning, ordering, and evaluating police referrals at the institutional level in the discussion about human right violations during crackdowns in Tijuana.

All respondents showed concern about being accused of kidnapping PWUD. Repeatedly, they emphasized that they referred people to drug centers in a voluntarily manner.

“There is a specific law that says that if you take people against their will you could be accused of kidnapping or illegal privation of liberty. So that’s why we don’t do it. It needs to be on a voluntary basis. It needs to be voluntary even if the person is high or is an addict” (Man, 42).

Finally, one respondent confirmed that law enforcers pushed people to get into drug centers, but he also criticized the citizens’ double moral standard that demands drug law enforcement without force and potential violation of human rights.

“Was it really a “clean up”? You just made it so that they were somewhere else and I don’t know what support was really offered to them. The citizens began to complain: “poor of them, their rights are being violated.” We have a society with a moral double standard. They want you to apply the law [quality-of-life ordinances], but they don’t want to give anything up in return, right? I mean, if you apply the law or you help them, you will be violating their rights. It’s natural! How can you help someone without violating their rights? You help them by forcing them! There are citizens who want you to do your job, to put them in jail, but when it comes to a cousin, a friend, a neighbor, their children: ‘not them officer, you’re so mean’” (Woman, 34).

## Discussion

In the present study, we found that MPOs reported that *Tijuana Mejora* was designed to avoid injuries in the rainy seasons, refer PWUD to drug centers, improve public security, and promote tourism. Prior to El Canal banishment, the government enforced soft and hard policies of exclusion by first offering and then demanding El Canal inhabitants to return to their places of origin or to get into shelters or drug centers. According to the overall narratives of the respondents, there was a contradictory feeling about the crackdown. On one hand, it was a successful police operation as they “cleaned”; El Canal. On the other hand, it was poorly implemented from a legal and human rights approach. For instance, the crackdown did not follow performance protocols (e.g., to avoid needle stick injuries while MPOs frisked PWUD at El Canal) and local judges did not follow the due process (e.g., being in the field hurriedly deciding where to refer hundreds of people).

For some MPOs, the main outcomes of *Tijuana Mejora* were a reduction in the perception of crime, traffic accidents on the nearby highways, and the eradication of visual signs of an open drug market. Participants considered they were doing right by breaking the vicious cycle of homeless PWUD by either convincing them or forcing them into drug centers. However, the fact that the city does not have enough infrastructure to provide evidence-based treatment was also a fact that participants considered when they were asked about the crackdown. Most local drug centers provide abstinence-only treatment, as opposed to evidence-based treatment, which is the standard care for people with drug use disorders (Meacham, Roesch, Strathdee, & Gaines, 2018; Schuckit, 2016).

Rafful and colleagues analyzed the *Tijuana Mejora* from the perspective of people who inject drugs (Rafful et al., 2019). In their study, participants reported intimidation and threats prior the banishment. These tactics created uncertainty about what would happen to them after the crackdown: to be taken into shelters as a preventive measure against floods, to be referred to drug centers, to be taken to police stations because of quality-of-life ordinances, or to be disappeared (i.e., never going back to El Canal, being permanently incarcerated or being killed). PWUD described being referred directly from El Canal to the drug centers or being taken from areas other than El Canal (e.g., Downtown) to El Canal and then to drug centers. This narrative ties with MPOs’ reports about municipal judges being transferred from the police stations to El Canal to facilitate and accelerate the referral process to shelters, drug centers, and the local jail. From both analyses, we can infer that El Canal became an open ‘court’ in which the municipal judges hurriedly defined the legal status of hundreds of individuals. However, it is not clear how the authorities decided (in an extremely short period of time) where to refer each person or how they distinguished between those who did not use drugs or require drug treatment and between those who did or did not have previous or pending criminal charges. It seemed that the local judges could not follow the due process. PWUD also informed that the verbal and physical violence that they experienced had increased between detentions and drug centers referrals (Rafful et al., 2019). However, in our study, MPOs did not report performing these abuses and many of them denied their participation in the crackdown and expressed their fear of being accused of kidnapping PWUD. Thus, *Tijuana Mejora* is far from being considered a harm reduction strategy, as other crackdowns abroad had suggested (Dixon & Maher, 2005).

In 2007, Mexico signed the Convention for the Protection of All Persons from Enforced Disappearance (UN, 2007). According to MPO responses, *Tijuana Mejora* violated its Article II as people were arrested, detained, and deprived of their liberty by MPOs with the direct support of the federal, state, and local governments followed by a refusal to acknowledge the deprivation of their liberty. In fact, the abduction and incarceration in drug centers was disguised as a drug treatment referral in which some PWUD were coerced to sign a consent admission to stay in drug centers (Rafful et al., 2019). Through MPOs and PWUD narratives, it has become evident that the Mexican authorities in the border region have territorially stigmatized the people living in El Canal. In fact, this crackdown was intended to lock PWUD in jail/shelters/drug centers and place them in a situation that they decided not to come back to El Canal.

Imprisonment is often used as a temporary solution to the growth of urban poverty. So, future studies may consider analyzing the parallels that exist between local jails and drug centers, as well as to examine policing practices in marginal spaces wherein homeless PWUD are being excluded (DeVerteuil, 2002; Stuart, 2014). Considering that shelter and drug center survival depends on their ability to attract and retain clients, further exploration is required to understand the partnership between law enforcement institutions, shelters, and drug centers. It is also necessary to study the tensions between different arms (e.g., health services and law enforcement) of the local government when designing and implementing policy focused on homeless PWUD. It is also recommended to include the perspectives of other key stakeholders such as local citizens, business owners, and NGOs’ into the current discussion (DeVerteuil, May, & Mahs, 2009).

The crackdown showed that the federal, state, and municipal governments were well aligned to aggressively and temporarily enforce the local law in Tijuana. As pointed out during the interviews, *Tijuana Mejora* was conducted in response to a federal government request to clean El Canal. Another participant stated that those people who did not want to be referred to drug centers were apprehended for violations to quality-of-life ordinances. In contrast, previous research has suggested the lack of coordination between the three levels of government to enforce the *Narcomenudeo* reforms in Tijuana (Beletsky et al., 2015; Morales et al., 2020). For instance, MPOs reported that *Narcomenudeo* reforms were created to be enforced by federal and state police officers, not by local ones (Morales et al., 2020). Consequently, further research needs to be done to characterize the mechanisms of coordination between the three levels of government and to clarify why the coordination was successful in enforcing the local ordinances but not the federal/state law on drugs, as the latter have more legal weight than the former.

The geographic displacement of PWUD, while harmful to the vulnerable populations involved, has little (if any) long term effect on the local drug market and drug consumption patterns. As in other open drug markets (e.g., Vancouver) (Kerr et al., 2005; Small et al., 2006), Tijuana's drug market was decentralized from El Canal to a larger number of locations over the city following the crackdown. Likewise, PWUD temporarily moved from El Canal to remote places, private venues (e.g., shooting galleries) or to less visible outdoor locations (e.g., hotels) to avoid law enforcers (Notimex 03/15/, 2015). However, a year later, in January 2016, it was reported that hundreds of people had escaped or been discharged from drug centers and some have since then moved back into El Canal storm drain tunnels to avoid being harassed by MPOs (Guerrero, 2016). Even at the time of the interviews, participants expressed having to face the unintended consequences of the dispersion of homeless people throughout the area. As the “coach roach effect” that a participant reported, police officers were enforcing quality-of-life ordinances in areas in which they did not use to do it before the crackdown. It would be important to analyze how these changes in the policing environment may have affected the rest of the population in Tijuana.

Worldwide, geographical dispersion of PWUD after crackdowns has been significantly associated with risky injection behaviors, risks of acquiring blood-borne infections, reluctance to access medical services, and strengthening of the illegal drug market and crime (Maher & Dixon, 2001a; Shepard & Blackley, 2005; Small et al., 2006; Wood et al., 2003). Therefore, it is also necessary to conduct further research on the impact of *Tijuana Mejora* or other police crackdowns on blood-borne infections transmission (Small et al., 2006), deterrence effect, drug market violence in the short- and long-term (more than 6 months) (Mazerolle et al., 2007), and human rights violations (Ediomo-Ubong, 2018). Only then may we evaluate the actual impact of such police operations in Tijuana and elsewhere.

It is also important to assess how the violence linked to organized crime alters the current relationship between MPOs and PWUD in Tijuana. MPOs have worked under uncertainty and terror as part of the “war on drugs”. In fact, MPOs synthesized their working conditions in three guarantees: “at any moment we can die, we can be fired and we can be imprisoned” (Contreras-Velasco, 2017). This characterization of MPOs’ living conditions adds more complexity to our understanding of police discretion and harassment when enforcing prohibitionist drug-related policies. MPOs may be perceived as both victimizers and victims. Thus, we need to clearly differentiate their knowledge, attitudes, and behaviors on drug law enforcement at the personal, peer, and institutional levels, as well as in relation to PWUD, drug dealers, and drug traffickers.

Unfortunately, police crackdowns such as *Tijuana Mejora* are pervasive municipal phenomena that regularly manifest in a variety of geographic settings and political contexts. For example, in August 2019, police in Boston, Massachusetts, conducted a crackdown on a high drug-use community near the South End. Police systematically arrested people, confiscated and destroyed property, and displaced homeless residents over a weeklong series of raids. *Operation Clean Sweep* has been highly criticized by human rights activists and public health professionals; however, it serves to demonstrate how contemporary zero-tolerance policing and crackdowns remain a key municipal strategy. Our analysis of police perspectives following the *Tijuana Mejora* crackdown sheds light on the primary agents involved in the conduct of police crackdowns and may be relevant for future interventions to align police strategy with public health priorities.

### Limitations

Our study has several limitations. First, most of the respondents reported not participating in *Tijuana Mejora*. However, all of them had a clear understanding of the police operation and opinions regarding the aims and effectiveness. Second, desirability bias may be present in the narration of their experience. Still, the findings are comparable to what other studies have previously reported about crackdowns around the world (Aitken, Moore, Higgs, Kelsall, & Kerger, 2002; Coomber et al., 2017; Cooper, Moore, Gruskin, & Krieger, 2005; Ediomo-Ubong, 2018; Maher & Dixon, 2001a; Sherman, 1990; Wood et al., 2004). Third, only narratives of MPOs were included in the analysis. To our knowledge, no previous study had given voice to police officers in the context of crackdowns that target homeless population in Tijuana or elsewhere in Latin America. Also, in Tijuana, the local police is more numerous than the state and federal police (Zepeda, 2009). Moreover, MPOs interact more with PWUD compared to state and federal police officers. Finally, the New Mexican Penal Justice System, which introduced oral procedures, came into force after the interviews were conducted. Therefore, MPO and PWUD interaction may have changed under the new legal regime. Consequently, our results may be a starting point to compare how the legal environment impacts on drug law enforcement in Mexico.

## Conclusions

Previous studies have described and analyzed state actions against vulnerable populations around the world (Eby, 2006; Green, 1995; Maher & Dixon, 2001b; Wood et al., 2004; Wood et al., 2017). However, much can still be learned from this line of research as security forces continue to systematically violate human rights of PWUD. If states keep using the same abusive techniques against PWUD, then the problematization of such instruments must remain in academic discussions. Unfortunately, government control mechanisms of PWUD may tend to be more violent over time if goals are not achieved. In Tijuana, federal, state, and local governments aligned in a large drug law enforcement action plagued by non-compliance with the law and security protocols to remove the PWUD from El Canal. This study advances the public health understanding of compulsory, abstinence-only drug centers as extensions of the public apparatus to imprison PWUD. Therefore, it is also important to problematize drug centers as central actors in the violation of PWUD’s human rights. It is also necessary to unveil formal and informal agreements between governmental and non-governmental actors in the provision of services to PWUD. A failure to do so prevents social justice progress in violent contexts such as the US-Mexico border region. Understanding dynamics between police officers and community stakeholders, including drug treatment centers, may also help improve non-compulsory police referrals to evidence-based drug treatment and harm reduction programs. This represents a potentially positive public health impact stemming from regular interactions between law enforcement and PWUD.

*Tijuana Mejora* showed that drug law enforcement in Tijuana is far from consistent with a health-based approach on the local drug policy. Participants recognized the limitations of the police operation in terms of drug treatment referral and crime reduction. Furthermore, MPOs were afraid of being accused of kidnapping after involuntarily referred hundreds of people that were living at El Canal. The crackdown was primarily intended to detain, displace, and lock homeless PWUD, not to assist and rehabilitate them. We identified a modality of enforcement in which a penal policy is disguised as a social one. Moreover, we identified two types of police referral to drug centers in Tijuana. The first resulted from permanent law enforcer’s discretion when enforcing quality-of-life ordinances’ (the main aim was to lock PWUD up in the local jail). The second was the main outcome of a crackdown designed at the institutional level to lock PWUD up in drug centers. We need to define how institutional violence is driven by the decisions of empowered individuals that generate public policy daily at the municipal, state, and federal levels instead of merely focusing on MPOs’ behaviors and attitudes. We also need to promote the alignment of federal, state, and local laws on drugs to emphasize the application of a balanced approach on security and health. Finally, it is necessary to strengthen police training on the promotion of police officers as recovery managers in rescue operations (e.g., overdose), referral to harm reduction services (e.g., methadone maintenance therapy), and drug treatment centers (e.g., evidence-based centers). SHIELD has begun a process of transformation in the Tijuana police force by promoting education programs that favor a balanced approach (security-health) in drug policy.

## Data Availability

The datasets used and/or analyzed during the current study are available from the corresponding author on reasonable request.

## Abbreviations

PWUD: People who use drugs
MPO: Municipal police officer

## Footnotes

1 Consistent with Rafful et al., 2019, we chose to use the term drug centers rather than drug treatment centers because they do not provide evidence-based treatment.

## References

[CR1] Aitken C, Moore D, Higgs P, Kelsall J, Kerger M (2002). The impact of a police crackdown on a street drug scene: Evidence from the street. The International Journal on Drug Policy.

[CR2] Albicker SL (2014). *Identidades narrativas y estigma: Deportados en El Bordo de Tijuana (narrative identities and stigma: Deportees in El Bordo de Tijuana)*.

[CR3] Albicker SL, Velasco L (2016). Deportación y estigma en la Frontera México-Estados Unidos: Atrapados en Tijuana. Norteamérica.

[CR4] Avalos, J. M., & Utley, N. G. (2014). *Aproximación al ánalisis de datos cualitativos con atlas ti. Material Didáctico del taller de Análisis Cualitativo con atlas ti [approximation to the analysis of qualitative data with atlas ti. Teaching material of the qualitative analysis workshop with atlas ti]*. Mexico: Master's program in cultural studies, El Colegio de la Frontera Norte, Tijuana.

[CR5] Banta-Green CJ, Beletsky L, Schoeppe JA, Coffin PO, Kuszler PC (2013). Police officers' and paramedics' experiences with overdose and their knowledge and opinions of Washington State's drug overdose-naloxone-good Samaritan law. Journal of Urban Health.

[CR6] Beletsky, L. 2016. Law enforcement, drugs and the 'Public Health' approach. Weekly Criminal Justice Newsletter. Accessed 05/02/2018.

[CR7] Beletsky, L., Lozada, R., Gaines, T., Abramovitz, D., Staines, H., Vera, A., Rangel, G., Arredondo, J., & Strathdee, S. A. (2013). Syringe confiscation as an HIV risk factor: The public health implications of arbitrary policing in Tijuana and ciudad Juarez, Mexico. *School of Law Faculty Publications, 90*(2), 284-298. 10.1007/s11524-012-9741-3 PMC3675719 22806453

[CR8] Beletsky, L., Macalino, G. E., & Burris, S. (2005). Attitudes of police officers towards syringe access, occupational needle-sticks, and drug use: A qualitative study of one city police department in the United States. *International Journal of Drug Policy, 16*(4), 267–274.

[CR9] Beletsky L, Rich JD, Walley AY (2012). Prevention of fatal opioid overdose. Journal of the American Medical Association.

[CR10] Beletsky, L., Wagner, K. D., Arredondo, J., Palinkas, L., Magis-Rodriguez, C., Kalic, N., Ludwig-Barron, N., & Strathdee, S. A. (2015). Implementing Mexico's "Narcomenudeo" drug law reform: A mixed-methods assessment of ealy experiences among people who inject drugs. *Journal of Mixed Methods Research*, 1–8.

[CR11] Bowling B (1999). The rise and fall of New York murder. Zero tolerance or Crack's decline?. British Journal of Criminology.

[CR12] Brouwer, K. C., Lozada, R., Weeks, J. R., Magis-Rodriguez, C., Firestone, M., & Strathdee, S. A. (2011). Intraurban mobility and its potential impact on the spread of blood-borne infections among drug injectors in Tijuana, Mexico. *Substance Use & Misuse*, 1–10. 10.3109/10826084.2011.632465 PMC3979331 22136446

[CR13] Brouwer KC, Rusch ML, Weeks JR, Lozada R, Vera A, Magis-Rodríguez C, Strathdee S (2012). Spatial epidemiology of HIV among injection drug users in Tijuana, Mexico. Annals of the American Association of Geographers.

[CR14] Brown R (2018). The Mexican drug war and early-life health: The impact of violent crime on birth outcomes. Demography.

[CR15] Caulkins JP (2002). *Law Enforcement's role in a harm reduction regime*.

[CR16] Chamayou G (2012). *Manhunts. A philosophical history*.

[CR17] Charmaz K, Denzin NK, Lincoln YS (2000). Grounded theory. *Handbook of qualitative research*.

[CR18] Cohen J, Csete J (2006). As strong as the weakest pillar: Harm reduction, law enforcement and human rights. The International Journal of Drug Policy.

[CR19] Contreras-Velasco O (2016). Vivir en los márgenes del Estado: Un estudio en la Frontera México-Estados Unidos (living on the margins of the state: A study on the Mexico-United States border). Región y Sociedad.

[CR20] Contreras-Velasco O (2017). Institución policial, violencia y cultura del terror en Tijuana (police institution, violence and terror culture in Tijuana). Revista Mexicana de Sociología.

[CR21] Coomber, R., Moyle, L., & Mahoney, M. K. (2017). Symbolic policing: Situating targeted police operations/crackdowns’ on street-level drug markets. *Policing and Society*, 18. 10.1080/10439463.2017.1323893.

[CR22] Cooper H, Moore L, Gruskin S, Krieger N (2005). The impact of a police drug crackdown on drug injectors' ability to practice harm reduction: A qualitative study. Social Science & Medicine.

[CR23] DeVerteuil G (2002). Homeless mobility, institutional settings, and the new poverty management. Environment and Planning A.

[CR24] DeVerteuil G, May J, Mahs J (2009). Complexity not collapse: Recasting the geographies of homelessness in a 'punitive age'. Progress in Human Geography.

[CR25] Dixon D, Maher L (2005). Policing, crime and public health: Lessons for Australia from the 'New York miracle'. Criminal Justice.

[CR26] DOF (2014). *Ley general de Salud*.

[CR27] Eby D (2006). The political power of police and crackdowns: Vancouver's example. The International Journal of Drug Policy.

[CR28] Ediomo-Ubong N (2018). Police crackdowns, structural violence and impact on the well-being of street cannabis users in a Nigerian city. International Journal of Drug Policy.

[CR29] Gaines T, Werb D, Arredondo J, Alaniz VM, Vilalta C, Beletsky L (2017). The spatial-temporal pattern of policing following a drug policy reform: Triangulating self-reported arrests with official crime statistics. Substance Use & Misuse.

[CR30] Glaser BG (2004). Remodeling grounded theory. Qualitative Social Research.

[CR31] Goetz B, Mitchell RE (2006). Pre-arrest/booking drug control strategies: Diversion to treatment, harm reduction and police involvement. Contemporary Drug Problems.

[CR32] Green L (1995). Cleaning up drug hot spots in Oakland, California: The displacement and diffusion effects. Justice Quarterly.

[CR33] Greene JA (1999). Zero tolerance: A case study of police policies and practices in new York City. Crime & Delincuency.

[CR34] Guerrero, J. 01/28/2016. "Tijuana migrants Hide in tunnels as police raids get deadly." *KPBS*, News. https://bit.ly/343Kfx8.

[CR35] Hughes CE, Stevens A (2010). What can we learn from the portuguese decriminalization of illicit drugs?. British Journal of Criminology.

[CR36] Hunter G, McSweeney T, Turnbull PJ (2005). The introduction of drug arrest referral schemes in London: A partnership between drug services and the police. The International Journal of Drug Policy.

[CR37] INEGI. 07/26/2017. Datos preliminares revelan que en 2016 se registraron 23 mil 953 homicidios (preliminary data reveal that in 2016 23 thousand 953 homicides were registered). Aguascalientes, México Instituto Nacional de Estadística y Geografía.

[CR38] Kelling, G. L., & Wilson, J. Q. (1989). Broken windows. The police and neighborhood safety. *Atlantic Monthly*, 29–38.

[CR39] Kerr T, Small W, Wood E (2005). The public health and social impacts of drug market enforcement: A review of the evidence. The International Journal of Drug Policy.

[CR40] Maher L, Dixon D (2001). The cost of cracdowns: Policing Cabramatta's heroin market. Current Issues in Criminal Justice.

[CR41] Maher L, Dixon D (2001). The cost of crackdowns: Policing Cabramatta’s heroin market. Current Issues in Criminal Justice.

[CR42] May T, Hough M (2001). Illegal dealings: The impact of low-level police enforcement on drug markets. European Journal on Criminal Policy and Research.

[CR43] Mazerolle L, Soole D, Rombouts S (2007). Drug law enforcement. A review of the evaluation literature. Police Quarterly.

[CR44] Meacham MC, Roesch SC, Strathdee SA, Gaines TL (2018). Perceived treatment need and latent transitions in heroin and methamphetamine Polydrug use among people who inject drugs in Tijuana, Mexico. Journal of Psychoactive Drugs.

[CR45] Morales, M., Baker, P., Rafful, C., Mittal, M. L., Rocha-Jimenez, T., Clairgue, E., Arredondo, J., Cepeda, J. A., Strathdee, S., & Beletsky, L. (2020). Conflicting Laws and Priorities as drug policy implementation barriers: A qualitative analysis of police perspectives in Tijuana, Mexico. *Journal of Drug Policy Analysis*Under Review. 10.1515/jdpa-2018-0014.

[CR46] Newburn T, Jones T (2007). Symbolizing crime control. Theoretical Criminology.

[CR47] Notimex. 03/15/2015. "Comerciantes piden seguimiento tras desalojo en Río Tijuana (Merchants ask for follow-up after eviction in Río Tijuana)." *Milenio Diario*, Estados Accessed 04/11/2015 https://goo.gl/taQNZ8.

[CR48] O'Neill KL, Lofton K, Lardas J (2019). Hunted. Predation and Pentecostalism in Guatemala. *New studies in religion*.

[CR49] Ortega-Granados LA (2017). Los Empresarios y Sus Prácticas de Movilidad Para Enfrentar la Violencia en el Espacio social Fronterizo de Tijuana. Asian Journal of Latin American Studies.

[CR50] Osorio J (2015). The contagion of drug violence. Spatiotemporal dynamics of the Mexican war on drugs. Journal of Conflict Resolution.

[CR51] Pinedo M, Burgos JL, Zuniga ML, Perez R, Macera CA, Ojeda VD (2015). Police victimization among persons who inject drugs along the US-Mexico border. Journal of Studies on Alcohol and Drugs.

[CR52] Piñera, D., and G. Rivera. 2013. Tijuana in history. Just Crossing the Border, Colección Divulgación Cultural. Distrito Federal: Secretaría de Educación Pública; Comisión Nacional de Cultura; Centro Cultural Tijuana.

[CR53] Polomarkakis KA (2017). Drug law enforcement revisited: The “war” against the war on drugs. Journal of Drug Issues.

[CR54] Rafful C, Medina-Mora ME, González-Zúñiga P, Jenkins JH, Rangel G, Strathdee SA, Davidson PJ (2019). "somebody is gonna get hurt": Involuntary drug treatment in Mexico. Medical Anthropology.

[CR55] Schuckit MA (2016). Treatment of opioid-use disorder. The New England Journal of Medicine.

[CR56] Shepard EM, Blackley PR (2005). Drug enforcement and crime: Recent evidence from New York state. Social Science Quarterly.

[CR57] Sherman LW (1990). Police crackdowns: Initials and residual deterrence. Crime and Justice.

[CR58] Shirk DA (2014). A tale of two Mexican border cities: The rise and decline of drug violence in Juárez and Tijuana. Journal of Borderlands Studies.

[CR59] Small W, Kerr T, Charette J, Schechter MT, Spittal PM (2006). Impacts of intensified police activity on injection drug users: Evidence from an ethnographic investigation. The International Journal of Drug Policy.

[CR60] Strathdee SA, Arredondo J, Rocha T, Abramovitz D, Rolon ML, Patino E, Rangel G, Olivarria HO, Gaines T, Patterson TL, Beletsky L (2015). *A police education programme to integrate occupational safety and HIV prevention: Protocol for a modified stepped-wedge study design with parallel prospective cohorts to assess behavioural outcomes*.

[CR61] Strauss A, Corbin J, Denzin K, Lincoln YS (1994). Grounded theory methodology. *Handbook of qualitative resesarch*.

[CR62] Stuart F (2014). From 'Rabble Management' to 'Recovery Management': Policing homelessness in marginal urban space. Urban Studies.

[CR63] Stuart F (2015). On the streets, under arrest: Policing homelessness in the 21st century. Sociology Compass.

[CR64] Suddaby R (2006). From the editors: What grounded theory is not. The Academy of Management Journal.

[CR65] UN, edited by Committee on Enforced Disappearances (2007). International convention for the protection of all persons from enforced disappearance.

[CR66] Velasco, L., & Albicker, S. (2013). *Estimación y caracterización de la población residente de "El Bordo" del canal del Río Tijuana (estimation and characterization of the resident population of "El Bordo" of the Tijuana river channel)*. Colegio de la Frontera, Tijuana. https://bit.ly/2xsQq38.

[CR67] Volkmann T, Lozada R, Anderson CM, Patterson TL, Vera A, Strathdee SA (2011). Factors associated with drug-related harms related to policing in Tijuana, Mexico. Harm Reduction Journal.

[CR68] Wacquant L (2001). The penalisation of poverty and the rise of neoliberalism. European Journal of Criminal Policy and Research.

[CR69] Watson TM, Bayoumi A, Kolla G, Penn R, Fischer B, Luce J, Strike C (2012). Police perceptions of supervised consumption sites (SCSs): A qualitative study. Substance Use & Misuse.

[CR70] Wood, E., Spittal, P. M., Kerr, T., Small, W., Tyndall, M. W., O'Shaughnessy, M. V., & Schechter, M. T. (2003). Requiring help injecting as a risk factor for HIV infection in the Vancouver epidemic. *Canadian Journal of Public Health*. September–October, *94*(5), 355–359. 10.1007/BF03403560 PMC6979944 14577743

[CR71] Wood E, Spittal PM, Small W, Kerr T, Li K, Hogg RS, Tyndall MW, Montaner JSG, Schechter MT (2004). Displacement of Canada’s largest public illicit drug market in response to a police crackdown. Canadian Medical Association Journal.

[CR72] Wood EF, Werb D, Beletsky L, Rangel G, Cuevas Mota J, Garfein RS, Strathdee SA, Wagner KD (2017). Differential experiences of Mexican policing by people who inject drugs residing in Tijuana and San Diego. The International Journal of Drug Policy.

[CR73] Zepeda G, Donnelly RA, Shirk D (2009). Mexican police and criminal justice system. *Police and public security in Mexico*.

